# Analysis of drug-induced adverse reactions affecting appetite and taste using the Japanese Adverse Drug Event Report Database

**DOI:** 10.1186/s13104-026-07734-5

**Published:** 2026-02-20

**Authors:** Mari Maese, Shingo Kondo, Yuka Sakatsume, Makoto Takagi, Yuta Yokoyama, Hiroki Iwata, Noriko Kobayashi, Katsunori Yamaura

**Affiliations:** 1 Division of Social Pharmacy, Center for Social Pharmacy and Pharmaceutical Care Sciences, Faculty of Pharmacy, Keio University, Tokyo, Japan; 2 Keio University Community Pharmacy, Tokyo, Japan; 3 Division of Pharmaceutical Care Sciences, Center for Social Pharmacy and Pharmaceutical Care Sciences, Keio University Faculty of Pharmacy, Tokyo, Japan

**Keywords:** Drug safety information, Decreased appetite, Ageusia, Dysgeusia, Taste disorder

## Abstract

**Objective:**

Pharmacotherapy is a potential risk factor for undernutrition; however, the association between individual drugs and nutrition-related adverse reactions remains unknown. We aimed to explore drugs affecting appetite and taste using the Japanese Adverse Drug Event Report (JADER) database.

**Methods:**

JADER reports (April 2004–January 2025) were analyzed for drug safety signals across seven Preferred Terms (PTs): “Appetite disorder,” “Decreased appetite,” “Abnormal loss of weight,” “Ageusia,” “Dysgeusia,” “Hypogeusia,” and “Taste disorder.” Signal detected drugs were classified by therapeutic category, and their package insert information was reviewed.

**Results:**

Among 20,638 nutrition-related adverse reaction reports, signals were detected for 54, 14, 24, and 13 drugs for “Decreased appetite,” “Ageusia,” “Dysgeusia,” and “Taste disorder”, respectively. No signals were detected for the other three PTs. Regarding therapeutic category, other hormone preparations were common for “Decreased appetite,” while anti-viral agents were common for “Dysgeusia” and “Taste disorder.” Of these, five and 15 drugs lacked descriptions in the package inserts regarding decreased appetite and taste-related adverse reactions, respectively.

**Conclusions:**

This study clarified drugs that may affect appetite and taste, providing a focused list for early detection. These findings may enhance pharmacovigilance practices and inform clinical approaches for managing drug-induced nutritional concerns.

**Supplementary Information:**

The online version contains supplementary material available at 10.1186/s13104-026-07734-5.

## Introduction

Undernutrition, which is defined as insufficient intake of essential nutrients, is associated with adverse health outcomes in older adults, including decreased quality of life (QOL) and increased risk of premature mortality. Undernourished individuals have a 1.62- to 4.41-fold higher mortality rates compared with well-nourished individuals [1]. Although undernutrition has multifactorial causes, pharmacotherapy is considered a potential risk factor. Drug-induced taste disorders and decreased appetite are considered potential contributors to nutritional deterioration in clinical settings [2]. More than 250 drugs have been reported to potentially affect nutritional status through these mechanisms [3, 4]. Decreased appetite is the most consistent risk factor for undernutrition in the elderly; further, approximately 20% of taste disorders are induced by drugs [5]. Accordingly, these adverse reactions can substantially impair the QOL by increasing the risk of weight loss and undernutrition [6, 7]. We previously analyzed nutrition-related incidents in community pharmacies using a nationwide database in Japan. This previous study highlighted the importance of continuous monitoring by community pharmacists and the provision of information to other healthcare professionals in order to prevent adverse reactions [8]. However, most existing evidence has been based on mechanistic insights or theoretical assumptions. Few studies have examined candidate drugs associated with nutrition-related adverse reactions using real-world data, leaving the specific drugs involved largely unclear and underscoring the need for further research.

Many countries have developed large-scale spontaneous reporting systems to facilitate early detection and trend analysis of drug-induced adverse reactions. Since 2004, the Pharmaceuticals and Medical Devices Agency (PMDA) has collected reports of adverse reactions related to both prescription and over-the-counter drugs in Japan [9]. The Japan Adverse Drug Event Report (JADER) database is maintained by the PMDA and is freely accessible to the public, with personal information being completely anonymized. Numerous studies have analyzed data regarding adverse events (AEs) using statistical methods to detect drug safety signals in pharmaceuticals. Commonly used frequentist metrics included the Reporting Odds Ratio (ROR) and Proportional Reporting Ratio (PRR) based on disproportionality analysis [10]. However, there are no studies analyzing drug-induced decreased appetite using spontaneous reporting databases. Previous studies using the JADER database related to taste disorders have generally used the broad term “Taste disorders,” missing specific subtypes such as ageusia and dysgeusia [11]. Additionally, previous studies using the Food and Drug Administration (FDA) Adverse Event Reporting System (FAERS) database have been limited to assessing antibiotic-induced taste and smell disorders [12]. There remain no comprehensive analyses specifically focusing on nutrition-related adverse reactions.

Therefore, we aimed to explore potential safety signals for drug-induced adverse reactions that may affect nutritional intake, specifically decreased appetite and taste disorders, using the latest available JADER database. These exploratory findings are expected to raise awareness about drug-induced nutritional decline and provide a foundation for appropriate pharmacotherapy for patients at risk of undernutrition.

## Methods

### Construction of the analytical data table

The JADER database was downloaded from the PMDA website (https://www.pmda.go.jp/safety/info-services/drugs/adr-info/suspected-adr/0003.html). We analyzed data available to the public as of May 2025 (accessed on May 12, 2025). Additionally, all reports published between April 2004 and January 2025 were included in this comprehensive analysis. The JADER dataset comprises four tables: (i) patient demographics (demo), (ii) drug information (drug), (iii) adverse events (reac), and (iv) primary illness (hist). These tables can be linked using case identification (ID) numbers assigned to each report. Since this study specifically focused on adverse reactions, we only utilized the drug information and adverse events tables. The drug information table contains information regarding three types of drugs: “suspected drugs”, which are considered to be associated with AEs; “concomitant drugs”, which are used at the time of AE occurrence; and “interaction drugs”, which are considered to interact with suspected drugs. Only the records of suspected drugs were extracted for analysis in order to ensure a strong association between the drugs and adverse reactions. Duplicate reports were identified and removed prior to linking datasets using the ID number given that duplicate records may exist for the same report with different prescription or event onset dates. In case multiple suspected drugs were listed in a single report, each drug was considered as a separate adverse reaction.

## Signal detection of suspected drugs

To extract specific AEs, we utilized the Japanese version of the Medical Dictionary for Regulatory Activities (MedDRA) version 28.0, adopting the Preferred Terms (PTs) recommended for JADER analysis from the perspective of timeliness [13] We used the following PTs (PT Codes): “Appetite disorder (10060961),” “Decreased appetite (10061428),” “Abnormal loss of w eight (10000159),” “Ageusia (10001480),” “Dysgeusia (10013911),” “Hypogeusia (10020989),” and “Taste disorder (10082490).” We focused on adverse reactions listed in drug package inserts. PTs related to decreased appetite, weight loss, and taste disorders were identified by reviewing package insert descriptions and selecting relevant MedDRA terms. Any reports containing these terms as registered AEs were extracted for analysis. Further, we used three pharmacovigilance indexes for signal detection: ROR, PRR, and the Chi-squared statistics (χ²). The criteria for signal detection were as follows: a signal was considered significantly only when both of the following criteria were met: (a) ROR: the lower bound of the 95% confidence interval (CI) > 1, and (b) PRR ≥ 2, χ² ≥4, and number of specific reports ≥ 3 [14–16]. All dataset processing and analyses were conducted using R Analytic Flow 3.3.1 (Ef-Prime, Inc., Tokyo, Japan; https://r.analyticflow.com/en/download/).

## Classification of signal detection drugs according to therapeutic category

Drugs meeting the criteria for signal detection were categorized based on the therapeutic category. Drugs were classified based on the “DIVISION 87 - DRUGS AND RELATED COMMODITIES” published by the Ministry of Internal Affairs and Communications. In order to exclude adverse reactions attributable to cancer-related conditions, we excluded drugs classified under therapeutic category codes beginning with “42,” which correspond to antineoplastic agents. Furthermore, for the signal-detected drugs, we confirmed whether decreased appetite or taste-related adverse reactions were described in the package inserts.

## Results

**Fig. 1:**
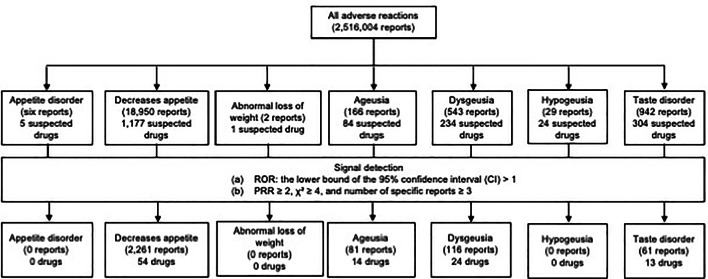
Flow chart of the analyses of data tables from the JADER Database The JADER dataset comprises four tables: (i) patient demographic (demo), (ii) drug information (drug), (iii) adverse event (reac), and (iv) primary illness (hist). These tables can be linked using a unique identification number assigned to each report Since this study specifically focused on adverse reactions, we only utilized the drug information and adverse events tables. The drug information table contains information regarding three types of drugs: “suspected drugs”, which are considered to be associated with AEs; “concomitant drugs”, which are used at the time of AE occurrence; and “interaction drugs”, which are considered to interact with suspected drugs. Only the records of suspected drugs were extracted for analysis in order to ensure a strong association between the drugs and adverse reactions

After removing duplicate records and merging the drug information and adverse event tables using ID numbers, 2,516,004 reports were obtained for analysis. Among them, 20,638 reports were identified by extracting those where any of the specified PTs were reported as AEs (Fig. 1). “Appetite disorder” was reported in six reports involving five suspected drugs. “Decreased appetite” was reported in 18,950 reports involving 1,177 different suspected drugs. “Abnormal loss of weight” was reported in two reports involving one suspected drug. “Ageusia” was reported in 166 reports involving 84 suspected drugs. “Dysgeusia” was reported in 543 reports involving 234 drugs. “Hypogeusia” was reported in 29 reports involving 24 suspected drugs. Finally, “Taste disorder” was reported in 942 reports involving 304 suspected drugs. In the signal detection analyses, 54 drugs (2,261 reports), 14 drugs (81 reports), 24 drugs (116 reports), and 13 drugs (61 reports) met the signal detection criteria for “Decreased appetite,” “Ageusia,” “Dysgeusia,” and “Taste disorder,” respectively. None of the drugs met the signal detection criteria for “Appetite disorder,” “Abnormal loss of weight,” or “Hypogeusia.”

Table 1 shows the therapeutic categories of the signal-detected drugs. There were 25 therapeutic categories of signal-detected drugs for “Decreased appetite”, with other hormone preparations (including antihormone preparations) being the most frequent (six drugs), followed by anti-viral agents (five drugs) and antiepileptics (five drugs). The detailed results of signal detection for “Decreased appetite” are presented in Supplementary Table S1. There were 42 signal-detected drugs for “Ageusia,” “Dysgeusia,” and “Taste disorder”, which belonged to 13, 16, and 10 therapeutic categories, respectively (Table 2). Among them, other agents for uro-genital and anal organs were common for “Ageusia” (two drugs), while anti-virus agents were common for both “Dysgeusia” (three drugs) and “Taste disorder” (four drugs). The detailed results of signal detection for each PT are presented in Supplementary Tables S2-S4. Eight drugs exhibited signals across multiple PTs, with their active ingredients being gefapixant citrate, terbinafine hydrochloride, zanamivir hydrate, azithromycin hydrate, nirmatrelvir & ritonavir, salmeterol xinafoate & fluticasone propionate, calcium folinate, and varenicline tartrate. Furthermore, among the 54 drugs with signals for “Decreased appetite,” 19 had package insert descriptions indicating as an adverse reaction. In contrast, only 3, 2, and 0 drugs showed such descriptions for “Ageusia,” “Dysgeusia,” and “Taste disorder,” respectively.

**Table 1:** Signal-detected drugs for “Decreased Appetite” categorized by therapeutic category

Therapeutic category	Number of drugs	Drug name	Total reports
Other hormone preparations (including antihormone preparations)	6	Dulaglutide (genetical recombination), tirzepatide, semaglutide (genetical recombination), osilodrostat phosphate, liraglutide (genetical recombination), mitotane	138
Anti-virus agents	5	Nirmatrelvir & ritonavir, ribavirin, amenamevir, glecaprevir hydrate & pibrentasvir, molnupiravir	507
Antiepileptics	5	Stiripentol, fenfluramine hydrochloride, rufinamide, topiramate, clobazam	95
Other biological preparations	4	Teceleukin (genetical recombination), interferon alfa (BALL-1), peginterferon alfa-2b (genetical recombination), interferon alpha (NAMALWA),	385
Agents affecting metabolism, n.e.c.	4	Pirfenidone, adalimumab (genetical resombination), nintedanib ethanesulfonate, anamorelin hydrochloride	153
Other agents affecting central nervous system	4	Riluzole, donepezil hydrochloride, memantine hydrochloride, rivastigmine	146
Opium alkaloids preparations	3	Morphine sulfate hydrate, hydromorphone hydrochloride, oxycodone hydrochloride hydrate	92
Antipyretics, analgesics and anti-inflammatory agents	3	Sulpiride hydrate & aminopropylone, tramadol hydrochloride, buprenorphine	61
Anti-tuberculous agents	3	Aluminoparaaminosalicylate calcium hydrate, ethionamide, delamanid	27
Antidotes	2	Calcium folinate, calcium levofolinate	472
Thyroid and para-thyroid hormone preparations	1	Teriparatide (genetical recombination)	34
Cardiotonics	1	Digoxin	23
Antidiabetic agents	1	Imeglimin hydrochloride	21
Purgatives and clysters	1	Lubiprostone	18
Antibiotic preparations acting mainly on acid-fast bacteria	1	Rifabutin	17
Other agents affecting digestive organs	1	Palonosetron hydrochloride	12
Antidiarrheals, intestinal regulators	1	Loperamide hydrochloride	10
Mineral preparations	1	Sodium ferrous citrate	10
Calcium compounds and preparations	1	Precipitated calcium carbonate & cholecalciferol & magnesium carbonate	9
Chinese meticines	1	Goshajinkigan	7
Antiparkinsonism agents	1	Levodopa & carbidopa hydrate & entacapone	7
Hypnotics and sedatives, antianxietics	1	Potassium bromide	5
Other cardiovascular agents	1	Ifenprodil tartrate	5
Psychotropic agents	1	Lisdexamfetamine mesilate	4
Diuretics	1	Acetazolamide sodium	3
Total	54		2261

**Table 2:** Signal-detected drugs for “Ageusia,” “Dysgeusia,” and “Taste Disorder” categorized by therapeutic category

Therapeutic category	Number of drugs	Drug name	Ageusia	Dysgeusia	Taste disorder	Total reports
Anti-virus agents	6	Ribavirin	−	17	−	54
		Nirmatrelvir & ritonavir	−	11	4	
		Zanamivir hydrate	3	−	4	
		Lamivudine	−	−	6	
		Laninamivir octanoate hydrate	−	5	−	
		Lopinavir & ritonavir	−	−	4	
Other agents for uro-genital and anal organ	3	Tamsulosin hydrochloride	8	−	−	16
		Silodosin	−	4	−	
		Solifenacin succinate	4	−	−	
Agents for hyperlipidemias	3	Atorvastatin calcium hydrate	7	−	−	14
		Pitavastatin calcium hydrate	−	4	−	
		Pravastatin sodium	−	3	−	
Hypnotics and sedatives, antianxietics	3	Brotizolam	−	6	−	12
		Triazolam	−	3	−	
		Zopiclone	−	3	−	
Other chemotherapeutics	2	Terbinafine hydrochloride	7	4	−	20
		Sulfamethoxazole & trimethoprim	−	−	9	
Vaccines	2	SARS-CoV-2 RNA vaccine	15	−	−	19
		Pneumococcal vaccine	−	4	−	
Antibiotic prepararions actiing mainly on gram-positive bacteria and mycoplasma	2	Azithromycin hydrate	3	3	5	16
		Clarithromycin	−	5	−	
Other agents affecting respiratory organs	2	Gefapixant citrate	4	3	−	13
		Salmeterol xinafoate & fluticasone propionate	−	3	3	
Synthetic antibacterials	2	Levofloxacin hydrate	6	−	−	11
		Linezolid	−	−	5	
Agents for peptic ulcer	2	Famotidine	7	−	−	10
		Esomeprazole magnesium hydrate	−	3	−	
Psychotropic agents	2	Paroxetine hydrochloride hydrate	−	6	−	10
		Sertraline hydrochloride	−	4	−	
Antidotes	1	Calcium folinate	−	5	4	9
Agents affecting metabolism, n.e.c.	1	Nintedanib ethanesulfonate	−	−	7	7
Agents for not mainly purpose therapeutic, n.e.c.	1	Varenicline tartrate	−	3	4	7
Cholagogues	1	Ursodeoxycholic acid	−	6	−	6
Vasodilators	1	Amlodipine besilate	6	−	−	6
Agents for liver disease	1	Monoammnonium glcyrrhizinate & glycine & L-cysteine	4	−	−	4
Agents for ophthalmic use	1	Latanoprost	−	4	−	4
Antihypertensives	1	Telmisartan	4		−	4
Other agents relating to blood and body fluids	1	Ethyl icosapentate	−	4	−	4
Anti-dermoinfectives	1	Fluconazole	−	−	3	3
Antihistamines	1	Chlorpheniramine maleate	3	−	−	3
Other agents affecting central nervous system	1	Suvorexant	−	−	3	3
Purgatives and clysters	1	Sennoside	−	3	−	3
Total	42		81	116	61	258

## Discussion

Since undernutrition is often asymptomatic and can be overlooked in clinical settings, there is a need for prompt detection and intervention. As aforementioned, the JADER database is the largest database for AEs in Japan and has been widely utilized in studies related to pharmacovigilance. Given that spontaneous reporting systems primarily capture serious AEs, this database serves as an important information source for pharmacoepidemiology.

There were 54 drugs that met the signal detection criteria for “Decreased appetite.” The most frequent therapeutic category was other hormone preparations (including antihormone preparations), which included six drugs. Among them, tirzepatide has been shown to decrease appetite in a dose-dependent manner, as documented in the Interview form containing clinical trial data. Glucagon-like peptide-1 (GLP-1) receptor agonists primarily exert their effects by suppressing appetite and increasing satiety via the hypothalamic GLP-1 receptor [17]. Among the signal detected drugs, stiripentol has already been reported to cause decreased appetite in clinical trials. In a Japanese Phase III clinical trial, decreased appetite was observed in 66.7% of the cases, with some cases progressing to undernutrition [18]. Stiripentol exerts its antiepileptic effects by enhancing gamma-aminobutyric acid (GABA) signaling, which is the principal inhibitory neurotransmitter. Dietary GABA may affect feeding behavior by activating vagal afferents [19]. Given that it is administered during or shortly after meals, this mechanism may result in decreased appetite. While such associations for tirzepatide or stiripentol are already reported through clinical trials, such signals serve as a continuous alert for healthcare professionals, emphasizing the persistence and clinical relevance of these adverse reactions in routine practice. Decreased appetite is not a mild or transient adverse event. Instead, it can result in clinically significant weight loss, which may increase the risk of geriatric syndrome among older adults. Accordingly, careful assessment of tolerability and continuous monitoring are essential during drug administration.

Notably, 14, 24, and 13 drugs met the signal detection criteria for “Ageusia,” “Dysgeusia,” and “Taste disorder,” respectively. Regarding the therapeutic categories, anti-viral agents were the leading category in both “Dysgeusia” (three drugs) and “Taste disorder” (four drugs). However, the package insert for ribavirin indicates that the incidence of adverse reactions is dependent on the combination of drugs used [20]. This suggests that concomitant medications may affect the detection of safety signals. Among the active ingredients, gefapixant citrate was signal detected for both “Ageusia” and “Dysgeusia.” These AEs have been reported to occur in 63.1% of patients, with the onset of most cases occurring within nine days of treatment initiation [21]. Gefapixant citrate, which is a selective P2 × 3 receptor antagonist, suppresses chronic coughing by inhibiting ATP signaling in sensory nerves; however, this mechanism of action may adversely affect taste. Compared with previous studies using the JADER database [11], our analysis not only confirmed the reproducibility of the previous research by encompassing it, but also newly detected six additional signals. Additionally, the novelty of our study lies in the integrated analysis of multiple AEs. Azithromycin hydrate, clarithromycin, and levofloxacin hydrate, were also signal detected in a previous study based on FAERS [12]. This concordance between independent reporting systems strongly supports a possible association of these agents with taste disorders. Accordingly, the Ministry of Health, Labor and Welfare has emphasized the importance of early detection and prompt management of drug-induced taste disturbances [22]. Although drug-induced taste disorders are generally mild, persistent cases may impair QOL. Therefore, close evaluation of medication regimens for older adults may facilitate the identification of the causes of taste disorders as well as improve both the quality and quantity of food intake.

Notably, this study included reports submitted after the approval of COVID-19 vaccines in Japan. The SARS-CoV-2 RNA vaccine was detected as a signal for “Ageusia.” As of April 2022, 4.3% of all reports were associated with COVID-19 vaccines, which suggests a potential reporting bias [23].

In conclusion, this study provides the first exploratory evidence linking individual drugs to nutrition-related adverse reactions by analyzing a large-scale spontaneous reporting database. These findings offer a focused list of potentially high-risk drugs, which may support the early detection of patients at risk of drug-induced nutritional decline as well as enhance clinical awareness.

### Limitations

This study has several limitations. First, spontaneous reporting is subject to reporting biases such as underreporting and influence by safety alerts or market trends. In particular, the choice of terms used by reporters affects the classification of AEs, and specific subcategories are likely to be underreported. Although multiple PTs exist within the same MedDRA hierarchy, we intentionally restricted the PT selection to prioritize signal specificity by excluding terms reflecting underlying psychiatric or metabolic pathologies (e.g., anorexia nervosa). While a broader PT set, as applied in previous pharmacovigilance [12] studies, may yield complementary findings. Our focused approach ensures the identification of signals directly relevant to clinical nutritional decline. Moreover, these systems tend to emphasize more severe AEs, relatively mild events may not be comprehensively captured. Therefore, reporting bias should be carefully considered, and their detection might be regarded as exploratory and potentially incomplete.

Second, individual patients cannot be uniquely identified across reports in the JADER database, our analysis was conducted using an event-based approach, which should be considered when interpreting the results. Third, to clarify the association between specific drugs and nutrition-related adverse reactions, it is important to use more detailed patient information, such as weight changes and nutritional indicators. Fourth, the JADER database does not differentiate between brand name and generic drugs, which impedes assessment of potential differences in AE reporting between drug types. Finally, signal detection does not prove causality; accordingly, our findings should be interpreted with caution.

## Supplementary Information

Supplementary Material 1.

## Data Availability

The datasets analyzed in this study are publicly available from the PMDA since the JADER database and can be freely downloaded from the official website.

## Abbreviations

AEs: Adverse events
PT: Preferred term
QOL: Quality of life
PMDA: Pharmaceuticals and Medical Devices Agency
MedDRA: Medical Dictionary for Regulatory Activities
JADER: Japan Adverse Drug Event Report
ROR: Reporting odds ratio
PRR: Proportional reporting ratio
