# Supplementary material for: Analysis of drug-induced adverse reactions affecting appetite and taste using the Japanese Adverse Drug Event Report Database
Source: BMC Res Notes. 2026 Feb 20;19:140. doi: 10.1186/s13104-026-07734-5 (PMC13032343; doi:10.1186/s13104-026-07734-5)
Supplement: Supplementary file 1 — Supplementary Material 1. [file 13104_2026_7734_MOESM1_ESM.docx]

Table S1. Statistical measures of ROR and PRR for “Decreases appetite.”

| Drug name | ROR (95% CI) | χ^2^ | PRR | Number of reports with adverse reactions | Total number of reports |
| --- | --- | --- | --- | --- | --- |
| stiripentol | 23.5 (16.1-34.2) | 564 | 20.1 | 32 | 212 |
| potassium bromide | 16.9 (6.66-42.9) | 52.8 | 15.1 | 5 | 44 |
| sulpiride hydrate & aminopropylone | 13.4 (5.36-33.8) | 41.5 | 12.3 | 5 | 54 |
| pirfenidone | 11.6 (8.09-16.5) | 282 | 10.7 | 33 | 410 |
| imeglimin hydrochloride | 10.7 (6.88-16.8) | 162 | 10.0 | 21 | 279 |
| aluminoparaaminosalicylate calcium hydrate | 9.42 (4.37-20.3) | 41.5 | 8.85 | 7 | 105 |
| fenfluramine hydrochloride | 8.91 (4.69-16.9) | 58.5 | 8.41 | 10 | 158 |
| rufinamide | 8.41 (3.69-19.2) | 30.1 | 7.97 | 6 | 100 |
| adalimumab (genetical resombination) | 6.94 (2.17-22.2) | 9.35 | 6.64 | 3 | 60 |
| dulaglutide (genetical recombination) | 6.73 (5.17-8.76) | 263 | 6.45 | 58 | 1,197 |
| tirzepatide | 6.35 (4.25-9.49) | 102 | 6.11 | 25 | 544 |
| calcium folinate | 6.34 (5.38-7.47) | 638 | 6.10 | 151 | 3,310 |
| lisdexamfetamine mesilate | 5.67 (2.08-15.4) | 10.6 | 5.48 | 4 | 97 |
| morphine sulfate hydrate | 5.63 (3.36-9.45) | 50.4 | 5.44 | 15 | 366 |
| semaglutide (genetical recombination) | 5.55 (3.80-8.10) | 95.7 | 5.36 | 28 | 694 |
| nintedanib ethanesulfonate | 5.27 (4.32-6.41) | 336 | 5.10 | 103 | 2,692 |
| osilodrostat phosphate | 5.17 (1.90-14.0) | 9.21 | 5.01 | 4 | 106 |
| rifabutin | 5.05 (3.11-8.20) | 49.3 | 4.90 | 17 | 461 |
| teceleukin (genetical recombination) | 5.01 (2.36-10.7) | 17.9 | 4.87 | 7 | 191 |
| interferon alfa (BALL-1) | 4.91 (2.31-10.4) | 17.4 | 4.77 | 7 | 195 |
| topiramate | 4.74 (3.20-7.01) | 70.3 | 4.61 | 26 | 750 |
| calcium levofolinate | 4.65 (4.15-5.20) | 869 | 4.52 | 321 | 9,549 |
| ethionamide | 4.63 (2.28-9.36) | 18.6 | 4.50 | 8 | 236 |
| acetazolamide sodium | 4.39 (1.39-13.9) | 4.66 | 4.28 | 3 | 93 |
| liraglutide (genetical recombination) | 4.36 (2.69-7.07) | 39.4 | 4.25 | 17 | 531 |
| hydromorphone hydrochloride | 4.13 (2.13-8.03) | 17.8 | 4.04 | 9 | 296 |
| oxycodone hydrochloride hydrate | 4.13 (3.25-5.26) | 153 | 4.04 | 68 | 2,242 |
| goshajinkigan | 3.98 (1.87-8.44) | 12.4 | 3.89 | 7 | 239 |
| peginterferon alfa-2b (genetical recombination) | 3.88 (3.49-4.31) | 734 | 3.80 | 362 | 12,838 |
| clobazam | 3.86 (2.50-5.96) | 40.5 | 3.78 | 21 | 738 |
| nirmatrelvir & ritonavir | 3.68 (2.56-5.30) | 54.4 | 3.61 | 30 | 1,104 |
| precipitated calcium carbonate & cholecalciferol & magnesium carbonate | 3.49 (1.80-6.76) | 13.2 | 3.43 | 9 | 349 |
| riluzole | 3.38 (1.50-7.60) | 7.60 | 3.32 | 6 | 240 |
| loperamide hydrochloride | 3.37 (1.80-6.32) | 14.0 | 3.31 | 10 | 401 |
| donepezil hydrochloride | 3.25 (2.62-4.04) | 125 | 3.20 | 84 | 3,496 |
| ifenprodil tartrate | 3.23 (1.33-7.85) | 5.5 | 3.18 | 5 | 209 |
| delamanid | 3.22 (1.82-5.71) | 15.8 | 3.17 | 12 | 503 |
| ribavirin | 3.20 (2.91-3.52) | 637 | 3.15 | 444 | 19,046 |
| tramadol hydrochloride | 3.17 (2.30-4.37) | 52.8 | 3.12 | 38 | 1,621 |
| mitotane | 3.13 (1.39-7.02) | 6.51 | 3.08 | 6 | 259 |
| buprenorphine | 3.09 (1.94-4.93) | 22.8 | 3.04 | 18 | 786 |
| palonosetron hydrochloride | 2.91 (1.64-5.15) | 12.9 | 2.87 | 12 | 556 |
| levodopa & carbidopa hydrate & ntacapone | 2.64 (1.25-5.57) | 5.44 | 2.60 | 7 | 357 |
| teriparatide (genetical recombination) | 2.56 (1.82-3.59) | 30.0 | 2.53 | 34 | 1,788 |
| digoxin | 2.49 (1.65-3.76) | 18.6 | 2.46 | 23 | 1,242 |
| memantine hydrochloride | 2.48 (1.63-3.78) | 17.6 | 2.45 | 22 | 1,191 |
| amenamevir | 2.40 (1.14-5.07) | 4.32 | 2.38 | 7 | 391 |
| lubiprostone | 2.40 (1.51-3.83) | 13.1 | 2.38 | 18 | 1,006 |
| rivastigmine | 2.39 (1.70-3.36) | 25.7 | 2.37 | 34 | 1,908 |
| interferon alpha (NAMALWA) | 2.33 (1.21-4.51) | 5.46 | 2.31 | 9 | 518 |
| glecaprevir hydrate & pibrentasvir | 2.32 (1.24-4.33) | 6.10 | 2.29 | 10 | 579 |
| molnupiravir | 2.23 (1.36-3.65) | 9.42 | 2.20 | 16 | 964 |
| sodium ferrous citrate | 2.22 (1.19-4.14) | 5.40 | 2.20 | 10 | 605 |
| anamorelin hydrochloride | 2.14 (1.26-3.64) | 7.30 | 2.13 | 14 | 875 |

Table S2. Statistical measures of ROR and PRR for “Ageusia.”

| Drug name | ROR (95% CI) | χ^2^ | PRR | Number of reports with adverse reactions | Total number of reports |
| --- | --- | --- | --- | --- | --- |
| gefapixant citrate | 1775 (624-5,051) | 4,754 | 1,593 | 4 | 39 |
| chlorpheniramine maleate | 118 (37.3-370) | 234 | 117 | 3 | 397 |
| monoammnonium glcyrrhizinate & glycine, aminoacetic acid & L-cysteine | 117 (43.1-315) | 339 | 116 | 4 | 537 |
| tamsulosin hydrochloride | 102 (49.8-207) | 661 | 101 | 8 | 1,262 |
| terbinafine hydrochloride | 51.8 (24.3-111) | 286 | 51.6 | 7 | 2,143 |
| solifenacin succinate | 47.7 (17.7-129) | 135 | 47.5 | 4 | 1,306 |
| zanamivir hydrate | 26.5 (8.49-83.2) | 49.4 | 26.5 | 3 | 1,747 |
| atorvastatin calcium hydrate | 23.9 (11.2-51.0) | 126 | 23.9 | 7 | 4,631 |
| telmisartan | 23.9 (8.84-64.4) | 64.5 | 23.8 | 4 | 2,604 |
| famotidine | 19.0 (8.89-40.4) | 97.3 | 18.9 | 7 | 5,837 |
| azithromycin hydrate | 14.5 (4.63-45.5) | 24.9 | 14.5 | 3 | 3,192 |
| amlodipine besilate | 11.8 (5.20-26.6) | 47.0 | 11.8 | 6 | 8,003 |
| levofloxacin hydrate | 10.3 (4.54-23.2) | 39.8 | 10.3 | 6 | 9,166 |
| SARS-CoV-2 RNA vaccine | 2.15 (1.26-3.65) | 7.33 | 2.15 | 15 | 111,160 |

Table S3. Statistical measures of ROR and PRR for “Dysgeusia.”

| Drug name | ROR (95% CI) | χ^2^ | PRR | Number of reports with adverse reactions | Total number of reports |
| --- | --- | --- | --- | --- | --- |
| gefapixant citrate | 388 (119-1,264) | 738 | 358 | 3 | 39 |
| laninamivir octanoate hydrate | 48.0 (19.8-116) | 182 | 47.5 | 5 | 492 |
| nirmatrelvir & ritonavir | 47.6 (26.1-86.7) | 442 | 47.1 | 11 | 1,104 |
| latanoprost | 26.2 (9.76-70.2) | 72.4 | 26.0 | 4 | 717 |
| ethyl icosapentate | 23.7 (8.85-63.6) | 65.0 | 23.6 | 4 | 791 |
| silodosin | 20.5 (7.66-55.0) | 55.4 | 20.4 | 4 | 913 |
| ursodeoxycholic acid | 19.9 (8.87-44.5) | 88.1 | 19.8 | 6 | 1,420 |
| zopiclone | 11.8 (3.78-36.7) | 19.6 | 11.8 | 3 | 1,189 |
| pitavastatin calcium hydrate | 11.0 (4.10-29.4) | 26.7 | 11.0 | 4 | 1,703 |
| sennoside | 10.3 (3.32-32.2) | 16.7 | 10.3 | 3 | 1,353 |
| brotizolam | 9.35 (4.18-20.9) | 36.3 | 9.34 | 6 | 3,007 |
| pravastatin sodium | 8.73 (2.80-27.2) | 13.4 | 8.71 | 3 | 1,603 |
| terbinafine hydrochloride | 8.72 (3.26-23.3) | 20.0 | 8.71 | 4 | 2,143 |
| salmeterol xinafoate & fluticasone propionate | 8.60 (2.76-26.8) | 13.2 | 8.59 | 3 | 1,626 |
| varenicline tartrate | 7.65 (2.46-23.8) | 11.2 | 7.64 | 3 | 1,829 |
| triazolam | 7.49 (2.41-23.3) | 10.9 | 7.48 | 3 | 1,868 |
| calcium folinate | 7.06 (2.93-17.1) | 20.1 | 7.06 | 5 | 3,310 |
| sertraline hydrochloride | 6.40 (2.39-17.1) | 13.1 | 6.39 | 4 | 2,917 |
| esomeprazole magnesium hydrate | 4.65 (1.49-14.5) | 5.29 | 4.65 | 3 | 3,005 |
| azithromycin hydrate | 4.38 (1.41-13.6) | 4.77 | 4.37 | 3 | 3,192 |
| ribavirin | 4.24 (2.62-6.87) | 37.6 | 4.24 | 17 | 19,046 |
| clarithromycin | 4.20 (1.74-10.1) | 9.08 | 4.19 | 5 | 5,565 |
| pneumococcal vaccine | 4.17 (1.56-11.2) | 6.67 | 4.17 | 4 | 4,474 |
| paroxetine hydrochloride hydrate | 3.99 (1.78-8.93) | 10.5 | 3.99 | 6 | 7,029 |

Table S4. Statistical measures of ROR and PRR for “Taste disorder.”

| Drug name | ROR (95% CI) | χ^2^ | PRR | Number of reports with adverse reactions | Total number of reports |
| --- | --- | --- | --- | --- | --- |
| lopinavir & ritonavir | 11.2 (4.17-29.9) | 27.3 | 11.1 | 4 | 964 |
| nirmatrelvir & ritonavir | 9.75 (3.64-26.1) | 23.1 | 9.71 | 4 | 1,104 |
| nintedanib ethanesulfonate | 7.01 (3.33-14.7) | 30.0 | 6.99 | 7 | 2,692 |
| lamivudine | 6.30 (2.82-14.1) | 21.5 | 6.29 | 6 | 2,562 |
| zanamivir hydrate | 6.15 (2.30-16.4) | 12.4 | 6.14 | 4 | 1,747 |
| suvorexant | 6.02 (1.93-18.7) | 8.0 | 6.00 | 3 | 1,338 |
| varenicline tartrate | 5.87 (2.20-15.7) | 11.6 | 5.86 | 4 | 1,829 |
| Fluconazole | 5.51 (1.77-17.1) | 6.98 | 5.50 | 3 | 1,461 |
| salmeterol xinafoate & fluticasone propionate | 4.95 (1.59-15.4) | 5.88 | 4.94 | 3 | 1,626 |
| azithromycin hydrate | 4.21 (1.75-10.1) | 9.15 | 4.20 | 5 | 3,192 |
| linezolid | 3.63 (1.51-8.74) | 7.02 | 3.62 | 5 | 3,699 |
| calcium folinate | 3.24 (1.21-8.66) | 4.13 | 3.24 | 4 | 3,310 |
| sulfamethoxazole & trimethoprim | 2.93 (1.52-5.66) | 9.50 | 2.93 | 9 | 8,256 |
